# Eating disorder patients with and without PTSD treated in residential care: discharge and 6-month follow-up results

**DOI:** 10.1186/s40337-023-00773-4

**Published:** 2023-03-27

**Authors:** Timothy D. Brewerton, Ismael Gavidia, Giulia Suro, Molly M. Perlman

**Affiliations:** 1 Department of Psychiatry and Behavioral Sciences, Medical University of South Carolina, Charleston, SC USA; 2 Timothy D. Brewerton, MD, LLC, Mount Pleasant, SC USA; 3 Monte Nido and Affiliates, Miami, FL USA; 4 Department of Psychiatry and Behavioral Health, Florida International University College of Medicine, Miami, FL USA

**Keywords:** Eating disorders, Trauma, Posttraumatic stress disorder (PTSD), Residential treatment, Depression, Anxiety, Quality of life, Outcome

## Abstract

**Introduction:**

We studied whether provisional posttraumatic stress disorder (PTSD) moderated discharge (DC) and 6-month follow-up (FU) outcomes of multi-modal, integrated eating disorder (ED) residential treatment (RT) based upon principles of cognitive processing therapy (CPT).

**Methods:**

ED patients [N = 609; 96% female; mean age (± SD) = 26.0 ± 8.8 years; 22% LGBTQ +] with and without PTSD completed validated assessments at admission (ADM), DC and 6-month FU to measure severity of ED, PTSD, major depressive disorder (MDD), state-trait anxiety (STA) symptoms, and eating disorder quality of life (EDQOL). We tested whether PTSD moderated the course of symptom change using mixed models analyses and if ED diagnosis, ADM BMI, age of ED onset and LGBTQ + orientation were significant covariates of change. Number of days between ADM and FU was used as a weighting measure.

**Results:**

Despite sustained improvements with RT in the total group, the PTSD group had significantly higher scores on all measures at all time points (*p* ≤ .001). Patients with (n = 261) and without PTSD (n = 348) showed similar symptom improvements from ADM to DC and outcomes remained statistically improved at 6-month FU compared to ADM. The only significant worsening observed between DC and FU was with MDD symptoms, yet all measures remained significantly lower than ADM at FU (*p* ≤ .001). There were no significant PTSD by time interactions for any of the measures. Age of ED onset was a significant covariate in the EDI-2, PHQ-9, STAI-T, and EDQOL models such that an earlier age of ED onset was associated with a worse outcome. ADM BMI was also a significant covariate in the EDE-Q, EDI-2, and EDQOL models, such that higher ADM BMI was associated with a worse ED and quality of life outcome.

**Conclusions:**

Integrated treatment approaches that address PTSD comorbidity can be successfully delivered in RT and are associated with sustained improvements at FU. Improving strategies to prevent post-DC recurrence of MDD symptoms is an important and challenging area of future work.

## Abstract (summary)

Posttraumatic stress disorder (PTSD) is common in patients with eating disorders and is associated with higher severity of symptoms and worse outcomes. However, this has not been studied extensively in patients admitted to higher levels of care, such as residential treatment. Using an integrated clinical approach based upon principles of cognitive processing therapy (CPT) and other evidence-based treatments, we studied outcomes at discharge and 6 months following discharge in 609 patients [96% female; mean age (± SD) = 26.0 ± 8.8 years; 22% LGBTQ +] with and without PTSD. All patients improved significantly and remained improved at follow-up compared to admission. However, all measured symptoms, including those of eating disorder, major depression, and state and trait anxiety, and a measure of quality of life, were worse in patients with PTSD at every time point (admission, discharge, and follow-up). The only significant worsening observed was for symptoms of major depression between discharge and follow-up. In conclusion, integrated treatment approaches that address PTSD and related problems can be successfully delivered in residential treatment and are associated with sustained improvements at 6 months following discharge. Improving strategies to prevent post-discharge recurrence of depressive symptoms is an important and challenging area of future work.

## Introduction

Posttraumatic stress disorder (PTSD) is a common comorbid condition in eating disorder (ED) patients, occurring in up to 50% of adults admitted to residential treatment (RT) [1–4]. An abundance of data from meta-analyses, national representative samples, clinical and community samples, and case control studies, have confirmed undisputable links between traumatic events and EDs, particularly for those with binge-type features [5–10]. Evidence also supports the contention that patients with significant traumatic histories and/or PTSD have distinct clinical features, including more severe ED symptoms, more suicidality, more anxiety and depressive symptoms, more experiential avoidance, and lower mindfulness [3, 4, 9, 11]. In addition, it is common for patients with EDs and PTSD (ED-PTSD) to have earlier ED onsets, more complex courses of illness, greater rates of dropout, and less favorable outcomes, particularly when the PTSD is not addressed [12–20]. Available evidence also suggests that patients with ED-PTSD may be more disinhibited, more impulsive, more dysregulated, and more predisposed toward retraumatization and resultant perpetuation of PTSD [8, 11, 21].

Furthermore, there is a paucity of available evidence about effective treatment approaches for ED-PTSD patients, especially those admitted to higher levels of care, such as RT. Although there are numerous evidence-based treatments (EBTs) for EDs and PTSD independently, there are limited findings on integrated treatment approaches for this challenging comorbidity despite the recognized clinical need [11, 19, 22]. Cognitive processing therapy (CPT) is an evidence-based, trauma-focused form of cognitive behavioral therapy that significantly reduces the symptoms of PTSD [23, 24]. Recent research has emerged showing that CPT may be effectively incorporated into an overall treatment program for ED-PTSD patients [20, 25–27]. Despite these developments, the therapy provided in these studies was delivered in an outpatient setting following a treatment period in a partial hospital program. In other trials, the ED was initially treated in an inpatient setting followed by a combination of CBT for relapse prevention and CPT for PTSD in a day hospital setting. The application of integrated treatment approaches in higher levels of care, such as RT, remains a major challenge to the field [25, 26, 28]. The traditional approach has been to address these conditions independently notwithstanding evidence that their development and perpetuation is intertwined [29–31].

In this report, we describe the development and implementation of an integrated treatment approach for ED-PTSD patients that was adopted for patients admitted to RT. In addition, we describe integrated treatment recommendations based on available literature and clinical experience.

The focus of this report is on admission (ADM) to discharge (DC) to 6-month follow-up (FU) outcome results from data generated from adults treated at ten RT sites across the United States over three years. The purpose of this study was to test whether PTSD moderated the course of symptom change from RT ADM to DC and then 6‐month FU using mixed models analyses. We hypothesized that all ED patients would respond to integrated RT, but that those with comorbid PTSD would show greater severity of ED, depressive, state-trait anxiety and poor quality of life symptoms at each time point of measurement. We also tested whether several factors previously shown to be associated with PTSD were significant covariates of symptom change across time, including ED diagnosis, ADM BMI, age of ED onset and LGBTQ + orientation.

## Methods

### Setting

Monte Nido & Affiliates (MNA) is a multi-site, multi-level comprehensive treatment program across 13 U.S. states for adolescent and adult individuals with severe EDs who require higher levels of care than outpatient.

### Ethics

This research project was approved by the Salus Institutional Review Board, and all participants gave written informed consent for the use of their assessment and follow-up results.

### Staff training

In an effort to improve patient outcomes, clinical and administrative leadership instituted a reassessment of programming in early 2016, which was followed by a comprehensive training program for staff on the interplay between trauma/PTSD and EDs. Training focused on teaching the principles of trauma-informed care and practice and the necessity for an integrated assessment and treatment protocol at higher levels of care. This was an evolution from the traditional sequential model of care when PTSD is identified [12, 32–34]. The MNA training program initially included a two-day training for all clinical directors, who are licensed senior therapists, by one of the authors (TB) in which the principles of assessing and treating ED-PTSD were presented. Previously published guidelines for initiating trauma treatment in ED-PTSD patients were described and are summarized elsewhere [11, 32, 34].

The second phase of preparation involved training of therapists in CPT, which included completion of an online course and a mandatory two-day therapist training by the originator, Patricia Resick, PhD at three national locations (2017) that was recorded. In addition, CPT manuals were provided for staff at all sites [23]. CPT was then integrated into an overall comprehensive ED-PTSD approach with ongoing supervision provided, initially by Dr. Resick for 20 sessions over 7 months for selected therapists, and then by others, including one of the authors (TB). The three primary CPT principles—(1) avoiding avoidance, (2) identifying stuck points, and (3) using Socratic questioning—were emphasized as essential clinical approaches that extended beyond the confines of CPT alone [23]. Rather than solely processing the traumatic event, CPT entails teaching skills for challenging distorted beliefs that one can use post-therapy. This approach makes it ideal for a time-bound therapeutic setting such as RT. In addition to an overall CPT-informed treatment approach, the decision to initiate the CPT protocol was determined on a case-by-case basis following consultation among the clinical team and under the direction of each site’s clinical director.

### Integrated treatment context

It is important to note that individual CPT sessions were delivered to patients within the safe and protected environment of RT, which incorporated a variety of EBT approaches as part of the overall treatment program. These included: (1) High levels of medical and psychiatric care, including psychopharmacologic interventions and 24-h nurse monitoring, in the comfort of a home-like setting, (2) Motivational enhancement approaches to support behavior change [35, 36], (3) Nutritional programming to meet differing nutritional needs [37], (4) Skills development through EBTs such as Dialectical Behavioral Therapy (DBT) and Cognitive Behavioral Therapy (CBT) [38–42], (5) marital/family therapies [43], and (6) yoga [44].

### Assessments

We have previously described the assessment instruments used in this study [2, 3]. These included: The Eating Disorder Examination Questionnaire (EDEQ) [45, 46], the Eating Disorder Inventory-2 (EDI-2) [47], the Patient Health Questionnaire (PHQ-9) [48], the Spielberger State-Trait Anxiety Inventory (STAI) [49], the Eating Disorder Quality of Life (EDQOL) scale [50], the Life Events Checklist for DSM-5 (LEC-5) [51, 52], and the PTSD Symptom Checklist for DSM-5 (PCL-5) [53].

Provisional diagnoses of PTSD according to the Diagnostic and Statistical Manual of Mental Disorders, Fifth Edition (DSM-5) were made via the Life Events Checklist for DSM-5 (LEC-5) for criterion A (endorsement of a life-threatening event that happened to the individual and/or was witnessed) and the PCL-5 for criteria B through E [54]. Inclusion criteria for being classified as having PTSD (PTSD +) were: (1) endorsement of at least one life-threatening event that happened to the individual and/or was witnessed, (2) having a PCL-5 total score of at least 33 or greater, and (3) endorsing each of the B through E DSM-5 criteria for PTSD as determined by PCL-5 responses [2, 3].

### Participants

There were 884 adults (≥ 18 years of age) with DSM-5 EDs entering and discharging from RT between January 1, 2018 and December 31, 2020, and 673 (76%) gave written informed consent. Of these, 609 (90.5%) completed ADM assessments and had a mean age (± SD) of 26.0 ± 8.8 years. Participants who were PTSD + (n = 261) accounted for 43% of the patients admitted to RT with complete data, while those who did not meet criteria for PTSD (PTSD-) (n = 348) accounted for 57%. Categorization by gender identity was as follows: 95.8% female, 3.3% male, 0.7% non-binary, 0.2% other. Classification by sexual orientation was done by self-report and was as follows: 78% heterosexual or straight, 12% bisexual, 5% gay or lesbian, and 5% other. Categorization by race was as follows: 93.1% white, 3.3% Asian, 2.2% black or African American, 1.1% American Indian/native Alaskan, and 0.4% native Hawaiian/other Pacific Islander. The majority (91.5%) of patients identified as of non-Hispanic origin. Regarding highest level of education attained, 10.8% completed high school, 3.5% had not, 4.6% had an associate degree, 33.3% completed some college, 21.0% had a bachelor’s degree, 5.3% completed some postgraduate education, 11.5% achieved a master’s degree or beyond, and 10% did not respond. Reported total household income was as follows: < $50,000: 37.2%; $50,000–$99,999: 11.6%; $100,000–$199,999: 4.3%; > $200,000: 0.8%; and 46% did not respond.

### Statistics

Analyses were conducted using SPSS version 27. Multivariate normality was assessed within the two groups: PTSD + and PTSD-. Rates of missing data varied by time point such that 6% of ADM data, 17% of DC data, and 54% of follow‐up data were missing. These percentages of missing data are expected in a treatment setting and are considered inevitable [55, 56]. Linear mixed effects models offer a simple alternative to handle missing data assuming missingness at random (MAR) without requiring imputations [57, 58].

Six multilevel models (MLMs), one for each primary variable of interest, were created to evaluate change across three time points (ADM, DC, FU) [59]. MLMs were chosen as the primary statistical method as they allow for flexibility in measuring multiple assessments over time for individual participants without the constraints imposed by other methods, such as repeated ANOVA. Furthermore, because MLMs measure the shape and rate of change over time for each participant’s data, they do not require a rigid data collection schedule. Additionally, MLMs are accommodating of missing data at various time points or data collected at differing spaced time points without relying on listwise deletion or imputation of data. The default of maximum likelihood estimation was used for all models run, slopes and intercepts. Time was measured in days to account for differences in time between ADM to RT and FU at 6 months.

Using MLMs permitted the assessment of change over our three study time points using linear or quadratic (linear) effects. Furthermore, given that the slope of data may accelerate or decelerate over time, it is also possible to log transform the time variable to improve overall model fit and allow for nonlinear modeling [56]. To address our research questions, we initially tested quadratic and log-transformed models. However, these models failed to converge thus demonstrating poor fit with the data. Using linear models demonstrated superior fit for the EDE-Q, EDI-2, EDQOL, STAI, PHQ-9 and PCL-5 models, thus these models were retained.

Finally, we controlled for body mass index (BMI), age at ADM, and primary ED diagnosis in all mixed models, as PTSD status was significantly associated with these characteristics (see Table 1). For each patient, primary ED diagnosis was dummy‐coded as one of the four categories: anorexia nervosa (restricting subtype), anorexia nervosa (binge–purge subtype), bulimia nervosa, and other diagnoses (primarily other specified feeding or eating disorder (OSFED) and binge eating disorder).

**Table 1:** Comparison of groups with provisional PTSD (PTSD +) and without provisional PTSD (PTSD-) on demographic characteristics and descriptive statistics

	PTSD −	PTSD +	X^2^	*p*
	n = 348	n = 261		
	n (%)	n (%)		
Race/ethnicity			7.37	.194
American Indian or Alaskan Native	5 (1.4)	1 (0.4)		
Asian	10 (2.9)	8 (3.1)		
Black or African American	4 (1.1)	8 (3.1)		
Native Hawaiian or other Pacific Islander	1 (0.3)	1 (0.4)		
White	300 (86.2)	212 (81.2)		
Missing	28 (8.0)	31 (11.9)		
ED diagnosis			21.65	** ≤ .001**
Anorexia nervosa-binge/purge	59 (17.0)	66 (25.3)		
Anorexia nervosa-restricting	158 (45.4)	71 (27.2)		
Bulimia nervosa	66 (19.0)	62 (23.8)		
OSFED and other	65 (18.7)	62 (23.8)		
Comorbid diagnoses				
Major depressive disorder	218 (68.1)	189 (73.3)	1.80	.179
PDD (dysthymia)	1 (0.3)	2 (0.8)	0.59	.440
Bipolar disorder	15 (4.7)	28 (10.9)	7.95	**.005**
GAD	211 (67.6)	143 (57.0)	6.76	**.006**
Obsessive–compulsive	20 (6.4)	27 (10.8)	3.44	**.045**
Substance use disorder	41 (13.1)	44 (17.5)	2.09	.092

	M (SD)	M (SD)	t	*p*
Age	25.70 (8.71)	26.63 (9.27)	− 1.26	.208
Body Mass Index (BMI)	20.20 (7.57)	22.47 (10.80)	− 3.02	**.003**

All statistically significant comparisons are in bold print for emphasis

The residual was weighted by the number of days between ADM and 6-month FU. ADM to FU is designated as Time 1, and DC to FU is designated as Time 2.

Several baseline covariates were included as simple main effects to be adjusted for, i.e., ED diagnosis, age at ADM, ADM BMI, age of ED onset, and LGBTQ + status, given their identified importance in previously published baseline results [2, 3, 60].

We tested for and established missingness at random (MAR) in two ways. First, we compared baseline variables between those with versus without missing data at ADM, DC and FU, and there were no significant differences in any of the measures. Secondly, in the same manner as Scharff et al. (2021), we performed a series of pattern mixture models to examine if a missing data pattern had a significant influence on the PTSD‐outcome association [58]. To do this, missing status was coded at the patient level, and the interaction between missingness, time, and PTSD diagnosis in mixed models analyses for each assessment of interest was conducted. In all cases, the addition of the missing data effects did not result in a significantly enhanced model fit. Taken together, these measures indicated that missing data did not significantly influence our results.

### Remission rates

To characterize the clinical significance of changes associated with treatment, we assessed the association between reaching subclinical threshold levels for the various measures and time points following treatment. Clinical thresholds were drawn from the relevant literature regarding clinical cutoffs for the EDE-Q, the PHQ-9, and the PCL-5 for diagnosing ED, major depressive disorder (MDD) and PTSD, respectively. Specifically, we used the following criteria to define subjects as below the clinical threshold or “in remission”:

1. EDE-Q: two global scale cutoffs were used, one < 1.55 [61] and the other < 2.3 [45];
2. PHQ-9: cutoff of < 10 [48];
3. PCL-5: total score cutoff of < 33 [62, 63];
4. STAI-S: score cutoff of ≤ 47.13;
5. STAI-T: score cutoff of ≤ 45.68. Although no clear clinical thresholds have been established for STAI-S and STAI-T, we used the mean plus one standard deviation (SD) that has been published for females ages 19–39 years [49].

## Results

### Baseline demographic and clinical characteristics

The demographic and clinical characteristics of the PTSD + and the PTSD- groups are shown in Table 1. There were no significant differences in age or race/ethnicity between the groups, although the PTSD + group was characterized by higher mean BMI and more frequent AN-BP, BN, OSFED, bipolar disorder and obsessive–compulsive disorder (OCD) diagnoses at ADM.

### Outcomes at each time point

Table 2 shows the descriptive statistics for all assessments at each time point. The effect sizes and 95% confidence intervals for each measure are shown for the time periods ADM to DC, DC to FU, and ADM to FU by PTSD group. The PTSD + group of patients reported greater severity of ED, major depressive and state-trait anxiety symptoms, as well as worse quality of life, not only at ADM but at all subsequent time points in comparison to the PTSD- group.

**Table 2:** Admission (ADM) to discharge (DC), DC to 6-month follow-up (FU), and ADM to FU descriptive statistics (mean (M), standard deviation (SD), effect sizes (Cohen’s d) and confidence intervals (CI) by provisional PTSD status

Symptom	Group	ADM, M(SD)	DC, M(SD)	FU, M(SD)	ADM-DC		DC-FU		ADM-FU	
					d (CI)	N	d (CI)	n	d (CI)	n
EDEQ global	PTSD +	**4.42 (1.22)**^**a**^	**2.71 (1.42)**^**b**^	**3.08 (1.74)**^**c**^	**1.32 (1.14, 1.50)**^**d**^	222	− 0.18 (− 0.38, 0.02)	96	**0.90 (0.69, 1.12)**^**f**^	115
	PTSD −	**3.48 (1.53)**	**2.00 (1.27)**	**2.34 (1.55)**	**1.12 (0.97, 1.26)**^**d**^	307	− 0.05 (− 0.22, 0.10)	142	**0.80 (0.62, 0.97)**^**f**^	164
EDI-2 total score	PTSD +	**123.08 (38.40)**^**a**^	**88.55 (43.00)**^**b**^	**92.99 (50.16)**^**c**^	**0.91 (0.75, 1.06)**^**d**^	214	0.00 (− 0.20, 0.20)	92	**0.74 (0.53, 0.95)**^**f**^	110
	PTSD −	**87.03 (39.76)**	**61.14 (36.62)**	**66.32 (41.86)**	**0.76 (0.63, 0.88)**^**d**^	298	0.02 (− 0.14, 0.18)	137	**0.63 (0.45, 0.79)**^**f**^	159
EDQOL total score	PTSD +	**2.37 (0.57)**^**a**^	**1.71 (0.77)**^**b**^	**1.64 (0.86)**^**c**^	**0.90 (0.74, 1.05)**^**d**^	216	0.10 (− 0.10, 0.30)	90	**0.83 (0.61, 1.05)**^**f**^	108
	PTSD −	**1.87 (0.63)**	**1.25 (0.72)**	**1.20 (0.81)**	**0.92 (0.78, 1.06)**^**d**^	293	0.16 (− 0.01, 0.33)	133	**0.74 (0.56, 0.91)**^**f**^	156
PCL-5	PTSD +	**52.72 (11.38)**^**a**^	**37.34 (18.65)**^**b**^	**40.24 (21.02)**^**c**^	**0.88 (0.72, 1.03)**^**d**^	220	− 0.09 (− .030, 0.10)	91	**0.68 (0.47, 0.89)**^**f**^	109
	PTSD −	**19.89 (12.97)**	**15.46 (14.08)**	**15.83 (15.21)**	**0.32 (0.20, 0.44)**^**d**^	295	0.01 (− 0.16, 0.18)	126	**0.22 (0.06, 0.38)**^**g**^	152
PHQ-9 depression	PTSD +	**19.71 (4.86)**^**a**^	**11.06 (6.57)**^**b**^	**13.72 (7.78)**^**c**^	**1.36 (1.17, 1.54)**^**d**^	221	− **0.30 (**− **0.51,** − **0.09)**^**e**^	93	**0.79 (0.58, 1.00)**^**f**^	111
	PTSD −	**14.24 (6.24)**	**7.62 (5.52)**	**9.60 (7.24)**	**1.09 (0.95, 1.24)**^**d**^	301	− **0.24 (**− **0.41,** − **0.07)**^**e**^	134	**0.61 (0.44, 0.78)**^**f**^	159
STAI state anxiety	PTSD +	**66.29 (9.72)**^**a**^	**56.27 (14.29)**^**b**^	**56.68 (16.13)**^**c**^	**0.80 (0.65, 0.96)**^**d**^	217	− 0.01 (− 0.21, 0.19)	91	**0.69 (0.48, 0.89)**^**f**^	109
	PTSD −	**57.13 (12.05)**	**47.52 (13.21)**	**48.92 (15.27)**	**0.68 (0.55, 0.80)**^**d**^	299	0.02 (− 0.14, 0.19)	133	**0.55 (0.38, 0.72)**^**f**^	157
STAI trait anxiety	PTSD +	**66.45 (8.35)**^**a**^	**57.58 (12.10)**^**b**^	**58.38 (14.25)**^**c**^	**0.81 (0.66, 0.96)**^**d**^	217	− 0.07 (− 0.27, 0.13)	91	**0.61 (0.40, 0.81)**^**f**^	110
	PTSD −	**56.83 (11.42)**	**49.54 (12.04)**	**51.24 (13.91)**	**0.68 (0.56, 0.81)**^**d**^	301	(− 0.14, 0.19)	133	**0.43 (0.27, 0.59)**^**f**^	157

All statistically significant comparisons are in bold print for emphasis

^a^Independent samples t-tests between PTSD + and PTSD- groups at ADM (*p* ≤ .001)

^b^Independent samples t-tests between PTSD + and PTSD- groups at DC (*p* ≤ .001)

^c^Independent samples t-tests between PTSD + and PTSD- groups at FU (*p* ≤ .001)

^d^Post-hoc t-tests between ADM and DC following mixed models analysis (*p* ≤ .001)

^e^Post-hoc t-tests between DC and FU following mixed models analysis (*p* ≤ .01)

^f^Post-hoc t-tests between ADM and FU following mixed models analysis (*p* ≤ .001)

^g^Post-hoc t-tests between ADM and FU following mixed models analysis (*p* ≤ .01)

Compared to ADM measures, both PTSD + and PTSD- groups displayed statistically significant improvements (*p* ≤ 0.001) in all clinical assessment measures at DC. Results also revealed statistically significant medium‐to‐large change effect sizes between ADM and DC for all measures except the total PCL-5 score in the PTSD- group (see Table 2). Improvements from ADM to DC were comparable for the PTSD + and PTSD- groups, although effect sizes were greater in the PTSD + group for all measures except EDQOL, in which they were nearly identical.

There were no significant differences in mean scores detected between DC to FU except higher major depressive symptoms (PHQ-9) at FU, which had small effect sizes for both the PTSD + and PTSD- groups (Table 2). Notably, confidence intervals for all measures overlapped zero regardless of PTSD status, which indicated the continuation of improvements achieved during RT through the FU time period.

### Remission rates

The proportion of patients with below‐threshold ED, major depressive, PTSD, and state-trait anxiety symptoms, or who attained remission, significantly differed by PTSD status at both DC and at FU (see Table 3).

**Table 3:** Rates of subthreshold symptom levels by provisional PTSD status at discharge (DC) and 6-month follow-up (FU)

	PTSD −	PTSD +	X^2^	*p*
	n (%)	n (%)		
*DC*				
EDE-Q below threshold of 1.55	118 (39.9)	54 (24.9)	12.607	**< .001**
EDE_Q below threshold of 2.3	173 (58.2)	85 (39.2)	18.256	** ≤ .001**
PCL-5 below threshold of 33	246 (86.0)	87 (40.5)	114.247	** ≤ .001**
PHQ-9 below threshold of 10	203 (69.8)	97 (44.9)	31.695	** ≤ .001**
STAI-S below threshold of 47.13	149 (51.4)	55 (25.9)	32.846	** ≤ .001**
STAI-T below threshold of 45.68	99 (34.0)	29 (13.6)	27.305	** ≤ .001**
*FU*				
EDE-Q below threshold of 1.55	63 (39.4)	28 (25.0)	6.115	**.013**
EDE_Q below threshold 2.3	86 (53.4)	40 (35.7)	8.328	**.004**
PCL-5 below threshold of 33	123 (82.6)	37 (34.9)	60.147	** ≤ .001**
PHQ-9 below threshold of 10	85 (54.5)	38 (35.2)	9.555	**.002**
STAI-S below threshold of 47.13	68 (43.9)	33 (30.8)	4.537	**.033**
STAI-T below threshold of 45.68	51 (32.9)	19 (17.8)	7.417	**.006**

All statistically significant comparisons are in bold print for emphasis

### Mixed models analyses

The results of the mixed model repeated measures analyses showed that the main effects of time (Time 1) and PTSD status were significant for all models tested, i.e., EDE-Q global score, EDI-2 total score, PCL-5 total score, PHQ-9 score, STAI-S score, STAI-T score, and EDQOL total score (Table 4). In the total group of participants, symptoms in all domains remained significantly lower (improved) from ADM to FU (Time 1) (*p* < 0.001). From DC to FU (Time 2), there was only slight worsening in the EDE-Q Global score (*p* = 0.017) but not in the EDI-2 total score, the STAI-S, the STAI-T, or the EDQOL scores. As noted in Table 2, EDE-Q global scores remained improved and significantly different from ADM at FU.

**Table 4:** Results of the mixed model repeated measures analyses for assessments of eating disorder, major depressive, state-trait anxiety symptoms and quality of life using linear time weighted by the number of days between admission and 6-month follow-up

Symptom	Estimate	SE	df	t	Sig (*p*)	95% CI (Lower–Upper)	
*EDE-Q global score*							
Intercept	1.633	0.570	679	2.864	**.004**	0.514	2.752
Time 1 (ADM-FU)	1.374	0.130	679	10.558	** ≤ .001**	1.118	1.630
Time 2 (DC-FU)	− .0321	0.135	679	− 2.385	**.017**	− 0.585	− 0.057
AN-BP	0.636	0.439	679	1.449	.148	− 0.226	1.499
AN-R	0.474	0.446	679	1.062	.288	− 0.402	1.350
BN	0.429	0.423	679	1.016	.310	− 0.400	1.259
OSFED	0.589	0.416	679	1.417	.157	− 0.227	1.406
Age at admission	− 0.002	0.007	679	− 0.280	.779	− 0.015	0.011
Admit BMI	0.025	0.009	679	2.699	**.007**	0.007	0.043
Age of ED onset	− 0.186	0.099	679	− 1.880	.061	− 0.380	0.089
LGBTQ	− 0.199	0.147	679	− 1.360	.174	− 0.487	0.089
PTSD	1.063	0.324	679	3.284	** ≤ .001**	0.427	1.698
PTSD × Time 1	0.001	0.003	679	0.224	.823	− 0.005	0.006
PTSD × Time 2	− 0.001	0.001	679	− 1.225	.221	− 0.004	0.001
*EDI-2 total score*							
Intercept	35.556	16.366	667	2.173	**.030**	3.422	67.692
Time 1 (ADM-FU)	27.347	3.737	667	7.317	** ≤ .001**	20.009	34.685
Time 2 (DC-FU)	− 4.647	3.865	667	− 1.202	.230	− 12.236	2.943
AN-BP	22.728	12.613	667	1.802	.072	− 2.037	47.494
AN-R	19.833	12.775	667	1.552	.121	− 5.251	44.917
BN	17.547	12.092	667	1.451	.147	− 6.196	41.290
OSFED	16.436	11.886	667	1.383	.167	− 6.903	39.774
Age at admission	0.101	0.198	667	0.512	.609	− 0.287	0.490
Admit BMI	0.872	0.268	667	3.251	** ≤ .001**	0.345	1.398
Age of ED onset	− 7.774	2.840	667	− 2.737	**.006**	− 13.351	− 2.198
LGBTQ	− 2.670	4.203	667	− 0.635	.525	− 10.924	5.583
PTSD	42.736	9.308	667	4.591	** ≤ .001**	24.460	61.013
PTSD × time 1	− 0.154	0.083	667	− 1.846	.065	− 0.318	0.010
PTSD × time 2	− 0.025	0.033	667	− 0.766	.444	− 0.090	0.040
*PHQ-9*							
Intercept	5.967	2.538	670	2.351	**.019**	0.984	10.949
Time 1 (ADM-FU)	5.081	0.578	670	8.788	** ≤ .001**	3.946	6.216
Time 2 (DC-FU)	-2.629	0.599	670	− 4.389	** ≤ .001**	− 3.806	− 1.453
AN-BP	3.566	1.954	670	1.825	.069	− 0.271	7.403
AN-R	3.399	1.980	670	1.717	.086	− 0.488	7.286
BN	2.917	1.873	670	1.557	.120	− 0.761	6.595
OSFED	3.519	1.841	670	1.911	.056	− 0.096	7.134
Age at admission	− 0.009	0.030	670	− 0.304	.762	− 0.069	0.051
Admit BMI	0.074	0.041	670	1.792	.074	− 0.007	0.156
Age of ED onset	− 0.918	0.439	670	− 2.092	**.037**	− 1.779	− 0.056
LGBTQ	0.396	0.648	670	0.611	.541	− 0.876	1.667
PTSD	5.404	1.434	670	3.768	** ≤ .001**	2.588	8.220
PTSD × time 1	0.002	0.013	670	0.172	.863	− 0.023	0.028
PTSD × time 2	− 0.005	0.005	670	− 0.969	.333	− 0.015	0.005
*STAI-state anxiety*							
Intercept	46.821	5.409	665	8.657	** ≤ .001**	36.201	57.441
Time 1 (ADM-FU)	8.866	1.237	665	7.167	** ≤ .001**	6.437	11.295
Time 2 (DC-FU)	− 1.621	1.280	665	− 1.266	.206	− 4.135	0.893
AN-BP	1.340	4.166	665	0.322	.748	− 6.840	9.520
AN-R	1.298	4.218	665	0.308	.758	− 6.985	9.581
BN	0.288	3.992	665	0.072	.943	− 7.551	8.127
OSFED	0.012	3.924	665	0.003	.998	− 7.694	7.718
Age at admission	0.005	0.065	665	0.073	.942	− 0.123	0.133
Admit BMI	0.096	0.089	665	1.084	.279	− 0.078	0.270
Age of ED onset	− 1.077	0.935	665	− 1.152	.250	− 2.913	0.759
LGBTQ	0.433	1.394	665	0.311	.756	− 2.304	3.170
PTSD	9.445	3.073	665	3.073	**.002**	3.410	15.479
PTSD × time 1	− 0.012	0.028	665	− 0.433	.665	− 0.066	0.042
PTSD × time 2	0.001	0.011	665	0.093	.926	− 0.020	0.022
*STAI-trait anxiety*							
Intercept	48.973	4.804	667	10.194	** ≤ .001**	39.541	58.406
Time 1 (ADM-FU)	6.431	1.097	667	5.861	** ≤ .001**	4.276	8.584
Time 2 (DC-FU)	− 1.691	1.137	667	− 1.487	.138	− 3.924	0.542
AN-BP	2.252	3.700	667	0.609	.543	− 5.014	9.518
AN-R	2.928	3.747	667	0.781	.435	− 4.430	10.286
BN	1.968	3.546	667	.555	.579	− 4.995	8.932
OSFED	1.0450	3.485	667	.300	.764	− 5.799	7.889
Age at admission	− 0.058	0.058	667	− 1.003	.316	− 0.171	0.055
Admit BMI	0.157	0.079	667	2.000	**.046**	0.003	0.311
Age of ED onset	− 1.843	0.831	667	− 2.220	**.027**	− 3.474	− 0.213
LGBTQ	1.056	1.233	667	.857	.392	− 1.365	3.477
PTSD	10.827	2.723	667	3.977	** ≤ .001**	5.481	16.173
PTSD × time 1	− 0.004	0.024	667	− .167	.867	− 0.052	0.044
PTSD × time 2	− 0.011	0.010	667	− 1.181	.238	− 0.030	0.008
*EDQOL total score*							
Intercept	1.205	0.286	664	4.210	** ≤ .001**	0.643	1.767
Time 1 (ADM-FU)	0.673	0.066	664	10.357	** ≤ .001**	0.546	0.801
Time 2 (DC-FU)	0.038	0.067	664	0.568	.571	− 0.094	0.170
AN-BP	0.0179	0.219	664	0.081	.935	− 0.413	0.449
AN-R	− 0.094	0.223	664	− 0.421	.674	− 0.531	0.343
BN	− 0.065	0.210	664	− 0.311	.756	− 0.478	0.347
OSFED	− 0.098	0.207	664	− 0.474	.636	− 0.504	0.308
Age at admission	− 0.003	0.003	664	− 0.952	.341	− 0.010	0.003
Admit BMI	0.012	0.005	664	2.482	**.013**	0.002	0.021
Age of ED onset	− 0.010	0.049	664	− 2.007	**.045**	− 0.196	− 0.002
LGBTQ	0.011	0.073	664	0.147	.883	− 0.132	0.153
PTSD	0.765	0.162	664	4.722	** ≤ .001**	0.447	1.084
PTSD × time 1	− 0.001	0.001	664	− 0.813	.416	− 0.004	0.002
PTSD × time 2	− 0.001	0.001	664	− 1.753	.080	− 0.002	0.0001

All statistically significant comparisons are in bold print for emphasis

*AN-BP* Anorexia nervosa, binge-purge type, *AN-R* Anorexia nervosa, restricting subtype, *BN* Bulimia nervosa, *OSFED* Other specified feeding and eating disorder, *BMI* Body mass index, *ED* Eating disorder, *LGBTQ* + Lesbian, gay, bisexual, trans, queer, plus, *ADM* Admission, *DC* Discharge, *FU* 6-month follow-up, *EDE-Q* Eating Disorder Examination—Questionnaire, *EDI-2* Eating Disorders Inventory—2, *PHQ-9* Patient Health Questionnaire—9, *STAI-S* Spielberger State-Trait Anxiety Inventory— State Scale, *STAI-T* Spielberger State-Trait Anxiety Inventory—Trait Scale, *EDQOL* Eating Disorder Quality of Life, *PTSD* posttraumatic stress disorder

Notably, the main effect of time (Time 2) was highly significant for the PHQ-9 model (*p* < 0.001), which indicated that symptoms of major depression significantly increased from DC to FU (Time 2). However, PHQ-9 scores remained significantly lower at FU compared to ADM (*p* < 0.001) (Table 2).

Several baseline covariates were included as simple main effects, i.e., ED diagnosis, age at ADM, ADM BMI, age of ED onset, and LGBTQ + status, given their identified importance in previously published baseline results [2, 3, 60]. There were several models in which certain baseline covariates were noted to be significant predictors of outcome.

Age at ADM was not found to be a significant covariate in any of the models, whereas the age of ED onset was a significant covariate in the EDI-2, PHQ-9, STAI-T, and EDQOL models such that an earlier age of ED onset was associated with a worse outcome.

ADM BMI was a significant covariate in the EDE-Q, EDI-2, and EDQOL models, such that higher ADM BMI was associated with a worse ED and quality of life outcome. A higher ADM BMI was associated with worse depression outcome (higher PHQ-9 scores) at a trend level, whereas neither state nor trait anxiety outcomes were significantly influenced by ADM BMI. Eating disorder diagnosis and LGBTQ + status were not significant covariates in any of the outcome models, although there was a trend that just missed significance for LGBTQ + to negatively impact EDI-2 total scores.

In each of the models, random intercept components were statistically significant, and there were no significant interactions between PTSD and time, although there were two trends: (1) an interaction between PTSD and Time 1 for EDI-2 total scores, suggesting greater change in EDI-2 total scores in the PTSD + group, and (2) an interaction between PTSD and Time 2 for EDQOL, also suggesting greater change in EDQOL total scores in the PTSD + group (see Table 4).

## Discussion

The trajectories of change for ED patients were similar for those with comorbid PTSD when compared to those without PTSD, although those with PTSD had significantly greater symptom severity at all time points in all domains. These results support the premise that multi-modal, integrated treatment approaches based on principles of CPT that address trauma and PTSD can be successfully delivered in RT to ED patients with PTSD and associated comorbidity.

Eating disorder symptoms as measured by EDE-Q global scale scores were noted to slightly worsen over time (Time 2) between DC and FU in the mixed model. However, this was not evident using EDI-2 total scores as a measure of ED symptoms. In addition, there was no significant difference noted in mean EDE-Q global scale scores between DC and FU, while those between ADM and FU remained highly significantly different from each other with large effect sizes. Our findings stand in contrast to those reported from other studies of ED patients treated in RT that did not receive trauma-focused treatment, such as CPT, and in which a significant worsening of ED symptoms at 6 months was noted [64]. This suggests that the addition of CPT may be an important treatment ingredient that produces more sustained improvements than other approaches. However, head-to-head comparisons would need to be completed to test this hypothesis.

Notably, the only model that revealed significant worsening between DC and FU (Time 2) was for the PHQ-9 score, a reliable measure of MDD symptoms [48]. Like other models, there was no PTSD by time interaction, indicating that worsening was present in both those with and without PTSD. This also stands in contrast to findings from other researchers who reported a different course of improvement between those with versus without PTSD [64].

Improving strategies to prevent post-DC recurrence of MDD symptoms is an important and challenging area of future work. MDD is very commonly associated with EDs, PTSD, and anxiety, and relapse is common [65–70]. Worsening or reemergence of major depressive symptoms may herald ED relapse and requires aggressive treatment that is ideally concurrent with treatment for ED and PTSD [71–73]. It is well established that depression is a risk factor for eating pathology and, conversely, eating pathology is a risk factor for depression [74–76]. Importantly, in a 22-year longitudinal follow-up study of ED patients, those who recovered were 2.2 times more likely to *not* have MDD [77]. Longer periods of follow-up after RT are indicated to confirm these results but are challenging to obtain. Nevertheless, these findings offer opportunities to explore available and emerging treatment modalities for MDD in the context of ED and perhaps PTSD. These may include more effectively delivered evidence-based psychotherapies, new psychopharmacologic agents, e.g., 5-HT4 receptor antagonists [78], combining other evidence-based psychotherapies and psychopharmacologic approaches [79], adjunctive utilization of neuromodulation, such as repetitive transcranial magnetic stimulation (rTMS) [80], deep TMS [81] or electroconvulsive therapy (ECT) [82], novel psychotropic- or psychedelic-assisted therapies, such as ketamine/esketamine, 3,4-methylenedioxymethamphetamine (MDMA), psilocybin, and ayahuasca [83–88], as well as deep brain stimulation (DBS) [89–91], although many of these newer approaches remain experimental and difficult to obtain. Apart from the clinical challenges, these findings offer new opportunities to better understand the underlying psychological and neuropsychobiological mechanisms mediating treatment response and refractoriness.

A unique finding from our study is that the age of ED onset was a significant predictor of response in several models, including those for EDI-2, PHQ-9, STAI-T and EDQOL. There was also a trend for an effect on EDE-Q. This indicates that early age of ED onset is a negative predictor of outcome in terms of ED, MDD, state anxiety symptoms and poorer quality of life. We have previously reported that early age of ED onset was associated with significantly more PTSD and greater severity of depression and state-trait anxiety as well as worse quality of life at ADM [60]. The results from this study indicates that early age of ED onset may also be associated with a poorer prognosis.

Along these same lines, we also found that ADM BMI was a significant factor influencing outcome, with higher ADM BMI predicting worse measures of ED (both EDE-Q and EDI-2), trait anxiety, and quality of life at FU. There was also a trend for a similar effect on MDD symptoms. These results are in keeping with the importance of tailoring interventions for ED patients with higher weights, which have often been under-recognized and under-treated [92].

Taken together, our results indicate that ED-PTSD patients can be successfully treated using a trauma-informed approach with CPT that is integrated with other concurrent EBTs. In our study ED-PTSD patients evidenced significant improvements in ED, PTSD, comorbid symptoms of anxiety and depression, and quality of life during their stay in RT. Effect sizes were noted to be large for all measures in patients with PTSD for the intervals ADM to DC and ADM to FU. For those without PTSD, effects sizes were large for EDE-Q global scores, EDQOL total scores, and PHQ-9 scores, and were medium for STAI-S and STAI-T scores for the same intervals. To our knowledge this is the first report showing that EDs and concurrent PTSD can be successfully treated concomitantly using CPT principles in RT.

Our findings are notable given the conventional “wisdom” in the ED field that it is best to refrain from trauma work while in intensive treatment settings and defer this to later outpatient treatment. This is often driven by not only a lack of expertise in trauma assessment and treatment, but also by fears of opening the proverbial Pandora’s box and making the ED worse [93]. Others may be reluctant to start a course of treatment that may not be completed, yet avoidance of trauma/PTSD treatment often results in relapse and another treatment “failure” [12, 19, 34, 94]. We agree with the premise that nutritional rehabilitation ideally comes first before intensive psychotherapy, but our results indicate that it is not necessary to complete nutritional stabilization before assessing and treating trauma and PTSD, particularly when ED-PTSD patients are willing and motivated to proceed with CPT [32, 34]. Concurrent, parallel, but interwoven, approaches to treatment, one for ED and one for PTSD, can be delivered during the same treatment course by the same providers/therapists.

Importantly, our results also suggest that completion of all twelve CPT sessions is unnecessary to achieve significant results. Data regarding the number of CPT sessions was not collected for all patients in this study. This is in keeping with previous findings that 58% of outpatients receiving a more flexible CPT protocol improve prior to completion [95]. Many therapists and programs may think that unless the trauma-focused treatment will be completed, it should not be initiated. Our results suggest differently and indicate that even some exposure to CPT and its principles is helpful, imparts a better understanding of the precipitating and/or perpetuating factors in EDs and comorbidities, and often instills hope of recovery to individuals who have experienced multiple traumas and suffered chronic EDs complicated by chronic PTSD. It is noteworthy that PTSD + patients demonstrated a 15.4-point mean decrease in PCL-5 total scores from ADM to DC, a difference that has been found to be a reliable change [96, 97]. The completed treatment of ED-PTSD to full remission is hypothesized to likely involve a long-term effort that extends to lower levels of care and beyond. Future studies using larger sample sizes, measures of the number of CPT sessions, and longer follow-up across levels of care are indicated.

The fact that 14% of PTSD- patients at DC and 17.4% at FU scored above threshold PCL-5 total score levels of ≥ 33 is worthy of comment. It may be that patients were either uncomfortable disclosing traumas on the LEC-5 and their sequelae upon ADM, or that with further nutritional rehabilitation and/or establishment of trust, they were subsequently able to acknowledge their PTSD symptoms. In addition, response prevention of ED symptoms that may have served to dampen emotional arousal and other PTSD symptoms may have also resulted in worsening for some patients.

### Strengths and limitations

There are several strengths of this study, including a satisfactory sample size and the utilization of reliable assessment instruments to quantify adverse life events, symptoms of PTSD, EDs, MDD, and state-trait anxiety, as well as measures of quality of life.

Despite these very promising findings, there are several limitations to this study. First, this is not a controlled trial but is rather an example of ongoing translational research that facilitates the connection between clinical science and its practical applications to people with EDs to improve health outcomes. There is a great need to develop integrated models to treat ED-PTSD, especially in higher levels of care [3, 4, 11, 18, 19, 26, 27, 64, 79].

Second, it is not clear how many CPT sessions were provided to each patient. It was at the discretion of the therapist and the clinical team as to whether CPT was offered, and some PTSD + patients did not choose to begin CPT due to a lack of readiness. Nevertheless, all patients regardless of PTSD diagnosis were exposed to a trauma-informed approach using psychoeducation and CPT principles [23]. Many of the patients who may not have completed the full protocol in RT stepped-down to the partial hospital program within MNA where CPT sessions could continue, and others were referred for continued CPT in lower levels of care outside of MNA. Patients were encouraged to continue the work they began in their future treatments, but this could not be measured.

Third, our sample lacked diversity in that over 90% of patients were white, non-Hispanic females and therefore is not a representative sample of EDs in the general population. However, it may represent those seeking care in RT centers in that patients from multiple locations and programs have reported high rates of traumatic life events, PTSD and psychiatric comorbidity [3, 4, 64, 98, 99].

Fourth, as previously noted, the attrition rate in our study was high with over 50% of data missing at FU. However, we have confidence that missing data did not significantly influence our outcome results in that we were able to establish MAR in two separate ways as noted previously.

In terms of the direction of future research, replication of these findings as well as outcome studies of integrated treatment protocols with follow-up periods longer than 6 months post DC are needed.

## Conclusions

Patients with EDs admitted to higher levels of care have high rates of trauma and PTSD, which is associated with symptom severity and worse quality of life. CPT has been utilized in outpatient settings successfully for ED-PTSD, but there are no data on its integration into RT.

This translational research demonstrates that integrated treatment for ED-PTSD using CPT in the context of other EBTs can be successfully implemented in RT. Patients with and without PTSD showed significant symptom improvements from ADM to DC and remained statistically improved at 6-month FU compared to ADM. Although the PTSD group had significantly higher scores on all measures at all time points, there were no PTSD by time interactions. Therefore, we did not find that PTSD significantly moderated changes in ED, MDD and state-trait anxiety symptoms or quality of life. Nevertheless, patients with PTSD were significantly less likely to attain subthreshold symptom levels for all measures at DC and at FU. The only significant worsening observed between DC and FU was with MDD symptoms, yet all measures remained significantly lower than ADM at FU. Improving strategies to prevent post-DC recurrence of MDD symptoms is an important and challenging area of future work. As this ongoing study continues, we are developing tools to assess the delivery and adherence of CPT in our programs. These findings may help to tailor more effective and integrated treatment approaches for individuals with ED-PTSD + admitted to higher levels of care.

## Data Availability

The data are proprietary and not available.

## Abbreviations

ADM: Admission
AN-BP: Anorexia nervosa, binge-purge type
AN-R: Anorexia nervosa, restricting subtype
BMI: Body mass index
BN: Bulimia nervosa
ED: Eating disorder
ED-PTSD: Eating disorder comorbid with PTSD
CBT: Cognitive behavioral therapy
CPT: Cognitive processing therapy
DBT: Dialectical behavioral therapy
DC: Discharge
EBT: Evidence-based treatment
ECT: Electroconvulsive therapy
EDE-Q: Eating Disorder Examination-Questionnaire
EDI-2: Eating Disorders Inventory-2
EDQOL: Eating Disorder Quality of Life
FU: 6-Month follow-up
LGBTQ +: Lesbian, gay, bisexual, trans, queer, plus
MDD: Major depressive disorder
MLMs: Multilevel models
OSFED: Other specified feeding and eating disorder
PHP: Partial hospital program
PHQ-9: Patient Health Questionnaire-9
PTSD: Posttraumatic stress disorder
RT: Residential treatment
rTMS: Repetitive transcranial magnetic stimulation
STAI-S: Spielberger State-Trait Anxiety Inventory-State Scale
STAI-T: Spielberger State-Trait Anxiety Inventory-Trait Scale
